# Applications of In Silico Models to Predict Drug-Induced Liver Injury

**DOI:** 10.3390/toxics10120788

**Published:** 2022-12-14

**Authors:** Jiaying Lin, Min Li, Wenyao Mak, Yufei Shi, Xiao Zhu, Zhijia Tang, Qingfeng He, Xiaoqiang Xiang

**Affiliations:** Department of Clinical Pharmacy and Pharmacy Administration, School of Pharmacy, Fudan University, Shanghai 201203, China

**Keywords:** drug-induced liver injury, in silico models, computer algorithm, machine learning, DILIsym, PBPK

## Abstract

Drug-induced liver injury (DILI) is a major cause of the withdrawal of pre-marketed drugs, typically attributed to oxidative stress, mitochondrial damage, disrupted bile acid homeostasis, and innate immune-related inflammation. DILI can be divided into intrinsic and idiosyncratic DILI with cholestatic liver injury as an important manifestation. The diagnosis of DILI remains a challenge today and relies on clinical judgment and knowledge of the insulting agent. Early prediction of hepatotoxicity is an important but still unfulfilled component of drug development. In response, in silico modeling has shown good potential to fill the missing puzzle. Computer algorithms, with machine learning and artificial intelligence as a representative, can be established to initiate a reaction on the given condition to predict DILI. DILIsym is a mechanistic approach that integrates physiologically based pharmacokinetic modeling with the mechanisms of hepatoxicity and has gained increasing popularity for DILI prediction. This article reviews existing in silico approaches utilized to predict DILI risks in clinical medication and provides an overview of the underlying principles and related practical applications.

## 1. Introduction

More than 30,000 drugs have been developed for diverse diseases, of which 1100 drugs could potentially cause liver injury. In the United Kingdom, the incidence of drug-induced liver injury (DILI) was reported as 13.9 per 100,000 inhabitants [1], while contemporary studies in China suggested a higher incidence of 23.8 per 100,000 persons with a different etiology from that of Western countries [2]. As one of the most severe adverse drug reactions (ADRs), DILI can damage the liver, causing acute liver failure (ALF), and fulminant hepatic failure that eventually requires a liver transplant or causes death [3,4,5]; however, due to the assorted clinical features and complex mechanisms of DILI, clinicians often fail to detect the condition early and miss the critical window to treat the patient effectively [6]. Incidents of DILI have been the major reason for regulatory bodies to decline new drug applications, or for pharmaceutical companies to modify dosing and regimens, declare prescription warnings, or withdraw the drug entirely from the market [7].

Currently, there are emerging preclinical human-relevant in vitro models used to evaluate the toxic injury of drug candidates to the liver. In these models, either single-cell type or multi-cell type assays can be performed [8]. The main difference between these two kinds of assays is the number of cell types used in the experiments. Only one of the primary human hepatocytes, immortalized liver-derived cell lines (e.g., HepG2, HuH7) or hepatocyte-like cells derived from stem cells, are generally used in single cell-type in vitro models, while multiple cell lines or multicellular co-culture systems are used as the representative of in vivo cellular behavior in multicell type assays [9]. Three-dimensional in vitro liver co-culture systems were also developed for the investigation of DILI, where cytochrome P450 (CYP450) inducibility and bile canaliculi-like structures are imitated [10]. Besides cytotoxicity assays, other in vitro assays are conducted to research the specific mechanism of potential hepatotoxicity. For example, the glucose-galactose assay and oxygen uptake assay can be conducted for the determination of mitochondrial injury [11]. Investigation for BSEP inhibition by drugs can be also a manner to evaluate DILI [12]. In addition, covalent binding assays and reactive metabolite trapping are used to detect the formation of reactive metabolites that can commonly cause liver injury [13].

Animal models also play an important role in the pharmacokinetics and toxicity researches of drug metabolism in vivo relevant to DILI. Several models of chimeric mice with humanized hepatocytes have been developed over the years, most of which require the damage of endogenous mouse hepatocytes followed by a transplant of human liver cells. Highly immunodeficient NOG mice (TK-NOG) are a humanized liver model expressing a herpes simplex virus type 1 thymidine kinase (HSVtk) transgene and mouse liver cells that were ablated after exposure to ganciclovir [14]. Humanized liver Fah−/−/Rag2−/−/Il2rg−/− (FRG) mice were developed by Azuma et al. by transplanting human hepatocytes into FRG mice whose endogenous hepatocytes were damaged due to the genetic block of the tyrosine catabolic pathway [15]. These humanized liver models exhibit comparable liver enzyme expression levels and activity to the donor livers [16], as an alternative tool to study the potential damage to the human liver of drug candidates. In addition to mouse models with humanized livers, several human CYP-transgenic mouse models have been generated. CYP450 humanization in mice can be achieved through the cross-breeding of a human CYP-transgenic mouse with a mouse-CYP knockout mouse or directly knocking in the human genes to replace the mouse genes [17]. However, CYP450 humanization in mice can only investigate the action of a single human CYP transgene on drugs, hence the limited significance of these models to human drug hepatotoxicity assessment [18].

Commonly used tests to assess DILI, including in vitro experiments or in vivo animal models mentioned above, are sometimes inaccurate when the data were extrapolated to humans. In addition, it is difficult to detect DILI during prospective clinical trials as the relatively low DILI occurrence rate compared to other serious adverse events (SAEs) requires a disproportionately large sample size for detection. Ethical considerations also forbid re-challenge tests for DILI [19,20]. Although an interactive software “Evaluation of Drug-Induced Serious Hepatotoxicity” (eDISH), released by the FDA in 2004, aims at monitoring and evaluating drug liver toxicity in clinical trials, it mainly depends on the acquired clinical data (e.g., serum level of alanine aminotransferase) obtained from each trial subject and it cannot predict drug hepatotoxicity before drugs are applied in humans [21]. Therefore, early diagnosis of DILI or the timely referral of patients is of great importance for a drug throughout its whole lifespan. In silico methods provide an effective approach to screening drugs that may cause hepatotoxicity. We aim to review the major underlying mechanisms of DILI and the in silico approaches to predict DILI risks, including computer algorithm models and the DILIsym model.

## 2. The Classification and Mechanisms of DILI

### 2.1. Classification

DILI is defined as a series of reactions triggered by exposure to any artificial or natural compound that leads to acute or chronic liver injury according to the course of the disease. It can also be divided into intrinsic DILI (InDILI) and idiosyncratic DILI (IDILI) based on the pathophysiological mechanism. Intrinsic liver injury is usually dose-dependent and can be predicted directly from human or animal models [22]. InDILI is relatively common and generally occurs within 1 to 5 days after supratherapeutic doses of the perpetrator drug are given. InDILI is typically observed as elevated hepatic serum aminotransferase or alkaline phosphatase (or both) without jaundice. On the other hand, IDILI is relatively rare and unpredictable, with acute hepatocellular hepatitis as the primary manifestation, and has a latency period ranging from 5 to 90 days [23]. Additionally, IDILI is less dose-dependent and has significant interindividual differences, posing difficulties in predicting injury occurrence through animal experiments [24]. In addition to InDILI and IDILI, cholestatic liver injury is also an important classification of DILI. It is caused by impaired biliary transport resulting in the accumulation of bile acids in the liver and systemic circulation [25].

### 2.2. Mechanisms

#### 2.2.1. Oxidative Stress

**Figure 1:**
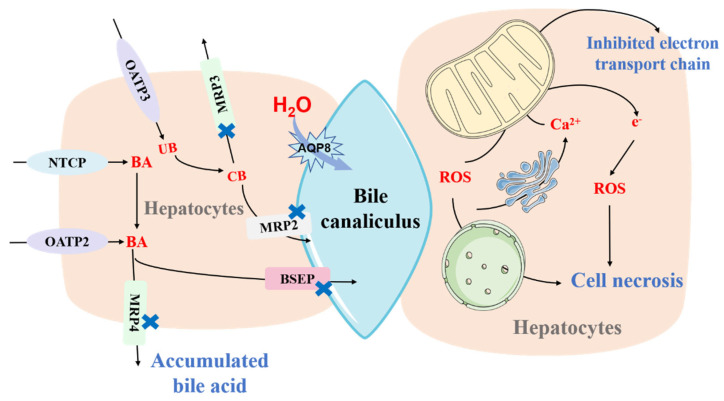
The mechanisms of drug−induced liver injury (DILI). Bile acid (BA) is transported into hepatocytes by NTCP and OATP2 and drained into bile canaliculus through MRP2 and BSEP. After entering into hepatocytes through OATP3, unconjugated bilirubin (UB) can be converted into conjunction bilirubin (CB), which exits hepatocytes via MRP2/3. Aquaporin−8 (AQP8) is responsible for maintaining the osmotic water permeability of the canalicular membrane. Inhibition of both MRP2/3/4, BSEP, and AQP8 by drugs can induce accumulation of bile acid and result in cholestasis. Inhibition of NTCP and OATP2/3 can induce increased plasma levels of bile acid. Some hepatotoxic drugs or their metabolites can be recognized as reactive molecules that present a similar action like reactive oxygen species (ROS), which damage mitochondria and cellular macromolecules, or directly impair mitochondrial function and cause the excessive generation of ROS, resulting in cell injury and death. NTCP, sodium taurocholate cotransporting polypeptide. OATP, organic anion transporter polypeptide. MRP, multidrug resistance-associated protein. BSEP, bile salt export pump.

Oxidative stress results from the excessive generation of reactive oxygen species (ROS), which are harmful to cells. For example, hydroxyl radicals can react directly with DNA components, phospholipids, and protein side chains to damage macromolecular structures and cause cell necrosis and apoptosis [26]. In addition, ROS can also change the functions of subcellular organelles, leading to alteration of the membrane permeability of the endoplasmic reticulum, sarcoplasmic reticulum, and mitochondrial reticulum to damage cells [27] (Figure 1). Some hepatotoxic drugs can be metabolized into reactive molecules with similar actions to ROS, which are known as reactive metabolites.

Acetaminophen (APAP), extensively used as an antipyretic, is a dose-dependent hepatotoxic drug. The main metabolic pathway of APAP at the therapeutic dose is phase II conjugation, where APAP is converted to nontoxic compounds including glucuronide derivatives (APAP-glu, 52–57%) and sulfate derivatives (APAP-sul, 30–44%). These polar metabolites are then excreted via urine. Only approximately 5% of APAP is oxidized by CYP450 enzymes in phaseImetabolism to form N-acetyl-P-benzoquinone imine (NAPQI), a strong oxidant, and subsequently conjugates with glutathione (GSH) [28]. However, a comparative proportion of APAP will undergo the phase I metabolizing pathway when phase II conjugating enzymes are saturated after excessive doses of APAP are given. This, in turn, produces more NAPQI and increases the need for GSH for conjugation [29]. Once GSH is exhausted, the reactive metabolite NAPQI will accumulate, leading to the damage of intracellular macromolecules and mitochondria [30]. Additionally, the depletion of GSH accelerates the formation of reactive oxygen and nitrogen in hepatocytes, further activating Kupfer and pleomorphic nuclear cells and leading to cell damage [31].

#### 2.2.2. Mitochondrial Toxicity

Mitochondria are essential organelles in eukaryotes. They are responsible for not only the production of energy—in the form of adenosine triphosphate (ATP)—but also the regulation of cellular biological activities including biosynthetic processes, calcium homeostasis, stress responses, and cell death [32]. Dysfunctional mitochondria would greatly disrupt normal physiological activities, leading to decreased ATP formation, ROS overproduction, and cell necrosis [33] (Figure 1). It has been reported that mitochondrial injury can occur during the development of DILI [34]. Many hepatotoxic drugs can disrupt mitochondria directly. Some drugs (such as rotenone, paroxetine, simvastatin, and tamoxifen) block electron transfers in the mitochondrial respiration chains [35], while others (such as troglitazone, amiodarone, valproic acid, tamoxifen, or glucocorticoids) diminish the β-oxidation of fatty acids in mitochondria through inhibiting acyl-CoA synthases, carnitine palmitoyl transferase I, or mitochondrial β-oxidation enzymes [36]. Additionally, some drugs can directly act on the mitochondrial genome and affect mitochondrial DNA (mtDNA) replication and translation, inducing mtDNA depletion that impedes oxidative phosphorylation [37]. Mitochondrial function can also be impaired following oxidative stress and abnormal cellular signal transduction induced by drugs. APAP can further activate Jun N-terminal kinase (JNK) signaling involving Apoptosis signal-regulating kinase 1 (ASK-1) and mitogen-activated protein kinase kinase kinase (MAPKKK), inducing the opening of the mitochondrial permeability transition pore and orchestrating the full collapse of mitochondrial function [38].

#### 2.2.3. Altered Bile Acid Homeostasis

The generation and secretion of hepatic bile depend on several transporters for bile acid transport and transmembrane water flow, such as sodium ion-dependent cholic acid transporters, bile salt removal pumps, and a number of water channels [39]. Cholestasis and mixed cholestasis are the primary manifestations of drug-induced liver injury in humans [25] that are caused by dysfunctional transporters involved in bile acid homeostasis (Figure 1). The bile salt export pump (BSEP), a biliary efflux transporter, plays a crucial role in driving intracellular organisms and exogenous substrates into bile. The inhibition of BSEP by some hepatotoxic drugs including cyclosporine, troglitazone, and bosentan can lead to the accumulation of intracellular bile acids and cholestatic damage in drug-induced liver injury [40]. However, it is challenging to predict liver damage from BSEP inhibition via preclinical animal models, thus many crucial mechanisms of drug-induced cholestasis remain poorly understood [41]. Besides BSEP, the sodium taurocholate cotransporting polypeptide (NTCP) is another predominant transporter located on the basolateral/sinusoidal membrane of hepatocytes that mediates the uptake of bile acids from the blood [42]. Kristina et al. demonstrated that some clinically hepatotoxic drugs (such as troglitazone and rifampin) could inhibit NTCP and obstruct the bile acids uptake into hepatocytes, which increased plasma levels of bile acids [43]. In addition, other biliary transporters like multidrug resistance proteins (P-glycoprotein and MDR3), multidrug resistance-associated protein 2/3/4 (MRP2/3/4), and organic anion transporting polypeptides 2/3 (OATP2/3) represent potential targets in drug-induced cholestasis [44].

Dysfunctional water channel proteins can also contribute to bile flow impairment. Aquaporin-8 (AQP8), a member of the aquaporin family of membrane channel proteins that facilitate osmotic pressure-driven water transport, is highly expressed on the canalicular membrane of hepatocytes [45]. Many studies showed that the downregulated expression of AQP8 was correlated with the reduced water permeability of the bile duct in a variety of cholestasis models [46], accounting for micellar concentrations of bile acids in the canaliculus and decreased choleresis [45].

#### 2.2.4. Innate and Idiosyncratic Immune Responses

Hepatic inflammation is the common manifestation of many liver diseases, including drug-induced hepatotoxicity [47]. The liver is the key organ of the immune system, and it is also the immune organ to show the resistance of lymphocytes [48]. Inflammatory phenotypes can be attributed to innate immune responses produced by Kupffer cells, monocytes, neutrophils, and lymphocytes. Activation of Kupffer cells and recruitment of macrophages and immune cells would lead to inflammation and injury due to increased cytokine release [49]. These events are significant factors in the initiation and maintenance of drug-induced liver injury and are especially important for the manifestation of IDILI.

Cytokines are important to regulate innate and specific immunity and also function as signals for potential danger. These messenger proteins bind to specific target cell receptors and can stimulate or inhibit cellular involvement in immune responses [30]. Some studies have reported that the decrease in interleukin-10 (IL-10) expression can cause severe consequences in IDILI patients due to the change of promoter and the reduction in serum eosinophil levels [50]. In addition, the drug and its metabolites can activate the immune response in the liver. For example, NAPQI, which accounts for the major hepatotoxicity risk of APAP, would trigger the broken balanced state of the innate immune system by activating signal transduction and transcription factor pathways involving inflammatory cascades following GSH depletion and covalent binding [51]. Previous studies have also shown that increased serum levels of high mobility group protein B1 (HMGB1)—a pro-inflammatory protein—could occur in patients who overdosed on APAP, suggesting that APAP-induced liver injury could be associated with innate immune inflammation [52].

## 3. The Prediction of DILI by In Silico Models

### 3.1. Knowledge-Based Prediction

**Figure 2:**
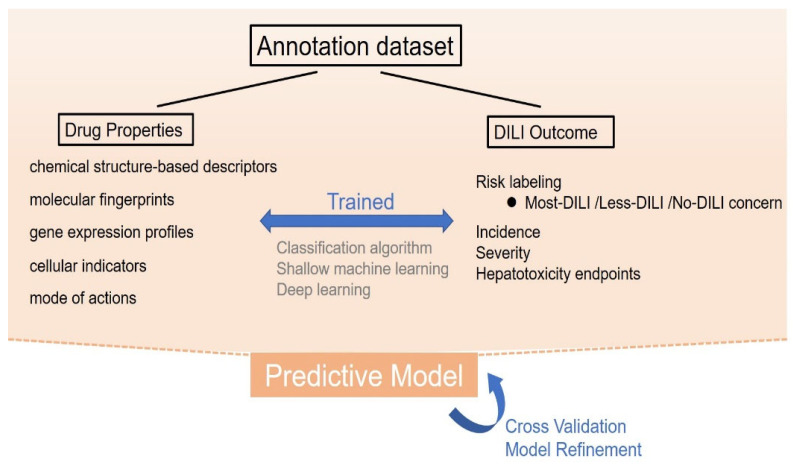
The process of knowledge-based prediction. In knowledge-based prediction, drug properties including molecular descriptors, molecular fingerprints, gene expression profiles, cellular indicators, and their mode of action are used to develop a certain relationship rule with the existing drug-induced liver injury (DILI) outcome, using classification algorithm, shallow machine learning, or deep learning methods. Through sufficient training, validation, and refinements, these models can be applied to predict the DILI risk of a new drug by preliminary properties.

Computer algorithms can be established to predict DILI based on a series of training data, which can be applied clinically or during drug development. To facilitate analysis, most DILI events can be divided into the input cause and output result. Input causes include the properties of drugs, such as chemical structures, gene expression profiles, and cell and tissue images. These properties are used to determine the probability of DILI occurrence. Output results could include in vitro and in vivo hepatotoxicity, changes in biomarkers, or clinical adverse events related to DILI [53]. By summarizing the rules between drug properties and DILI occurrence (see Figure 2) using computer algorithms followed by proper and sufficient training, clinicians can predict the DILI risk of a new drug by preliminary properties.

#### 3.1.1. Cheminformatics-Based Model

Chemical structures are commonly associated with the bioactivity of drugs and closely relate to the occurrence and severity of DILI. As a result, the development of Quantitative Structure-Activity Relationship (QSAR) via in silico models plays an important role in the prediction and assessment of DILI. In QSAR, the structure of each chemical compound will be treated as a vector known as a molecular descriptor. Then the functional relationship between molecular descriptors and a DILI-related biological activity of molecules (represented by a scalar) will be constructed [54]. Different chemical structure-based descriptors have been proposed, ranging from those with simple characteristics (that is molecular weight and number of carbon atoms) to sophisticated encodings typically referred to as “molecular fingerprints” [55].

There are different computational programs to process the input information, which can be divided into explicitly coded decision rules and implicitly defined rules. Explicitly coded decision rules are commonly used when expert toxicology or hepatotoxicity knowledge is available, in which the assessment of hepatotoxicity is determined by a fixed classification algorithm based on chemical structures [56]. However, most statistical models use implicitly defined rules, namely machine learning to achieve the same outcome. The primary algorithms typically can be trained with the ultimate decision rules obtained with optimization techniques [57].

##### Expert Knowledge Approaches

With the expert knowledge approaches (explicitly coded decision rules), known drug information is used to identify specific fragments of molecules that are associated with DILI. These are generally called structural alerts [58]. Egan et al. developed 74 computational alerts based on the structural and mechanistic information of 244 molecules using the Vertex cheminformatics platform (VERDI) that forms one of the published structural alert techniques. Of the 74 structural alerts, over 80 percent were related to the functional groups that are mainly converted to reactive toxic metabolites [59]. Derek for Windows is another knowledge-based tool for predicting toxicity, covering carcinogenicity, mutagenicity, skin sensitization, hepatotoxicity, and reproductive toxicity [60]. Greene et al. collected over 1266 chemicals and developed structure−activity relationships as structural alerts using Derek for Windows. The external evaluation of this model achieved an overall concordance of 56%, specificity of 73%, and sensitivity of 46% [61].

Besides structural alerts, some classification algorithms are developed to utilize existing drug knowledge for the DILI judgment. For example, Zhu and Kruhlak proposed a scoring rule for post-marketing DILI data of 2029 drugs with 13,555 drug-adverse event pairs and classified them as DILI-positive or -negative according to their respective scores [62].

##### Machine Learning Approaches

As mentioned above, the decision-making algorithms of machine learning (ML) are implicitly defined and typically obtained by algorithm optimization. Machine learning can be further classified as shallow ML methods (numerous naive Bayes classifiers, k-nearest neighbors, support vector machines, and random forests) and deep learning methods based on the so-called deep artificial neural networks with at least two hidden layers [63].

Due to their good accuracy, there are increasing numbers of predictive statistical models using machine learning methods. Chen et al. established a QSAR model using the Decision Forest algorithm, to calculate molecular descriptors from 2D chemical structures by Mold2. The model was trained by 197 drugs and assessed by 3 validation data sets with an overall estimated accuracy of 73.6% in high-confidence therapeutic subgroups [64]. Zhang et al. developed a computational model using human datasets and the Naive Bayes classifier approach. The structural features of various compounds were analyzed using 1D descriptors, AlogP, molecular properties, molecular property counts, surface area and volume, topological descriptors, and the extended connectivity fingerprints (ECFP), with overall prediction accuracy, sensitivity, and specificity of 94.0%, 97.1%, and 89.2% for the training set, respectively [65]. Liu et al. employed the Support Vector Machine (SVM) and random forest (RF) algorithms with ECFP4 fingerprints, Mordred molecular descriptors, and the predicted protein targets as chemical structure-derived descriptors for developing DILI classifiers [66].

Deep learning algorithms are increasingly popular in the prediction of DILI. Xu et al. constructed the DILI prediction model using the undirected graph recursive neural networks (UGRNN) method, relying only on a few suitable molecular descriptors with suitable representations that are learned automatically from the data. The DL-Combined model, which performed better than previous DILI prediction models, was trained on 475 drugs and predicted 198 drugs with 86.9% accuracy, 82.5% sensitivity, and 92.9% specificity [67]. Nguyen-Vo et al. used convolutional neural networks combined with molecular fingerprint-embedded features to screen DILI compounds and obtained an average accuracy of 89% [68]. Li et al. employed five conventional ML algorithms, including K-nearest neighbor (KNN), logistic regression (LR), SVM, RF, and extreme gradient boosting (XGBoost) in a three-layer neural network based on the Mold2 descriptors for the development of the DeepDILI model. The model was ultimately used to predict any DILI concern for potential 2019-nCOV treatments [69].

#### 3.1.2. Bioactivity-Based Model

To complement and improve chemical structure-based models, additional information in the form of biological drug properties such as gene expression data (i.e., genomic biomarkers for DILI prediction) and cellular indicators are necessary. Most gene expression data are accessible in some large-scale biomedical datasets such as the Connectivity Map (CMap) project, which is a collection of transcriptional expression data derived from cultured human cells treated with various compounds [70] and Genomics-Assisted Toxicity Evaluation System (TG-GATEs), another large toxicogenomics database with higher diversity of data structures [71]. The gene expression profile can be regarded as one of the drug features and correlated to hepatotoxicity outcomes for the prediction of DILI. Liu et al. collected different types of drug features, including chemical fingerprints, molecular descriptors, binding proteins, gene expression, therapeutic classifications, and different DILI endpoints such as liver failure, jaundice, biomarker increase, hepatomegaly, and hepatitis, and used these data to train logistic regression and random forest classifiers. The resultant areas under the receiver operating characteristic curve (AUC) were approximately 0.8 for certain DILI endpoints indicating that such a combination generally improved the model performance compared to only using a single feature [72]. Li et al. developed an eight-layer Deep Neural Network (DNN) model for DILI prediction using transcriptomic profiles of human cell lines and the model also achieved a comparative AUC of 0.798 for the independent validation set [73].

In addition, in vitro indicators determined by imaging assays can be a feature of chemical compounds, such as mitochondrial damage, oxidative stress, and intracellular glutathione. For example, Zhu et al. used human hepatocyte imaging assay technology (HIAT) descriptors that included several biochemical indicators (e.g., lipids and glutathione) of 156 DILI-positive and 136 DILI-negative compounds to build a DILI predictive model. Compared to the chemical structure-based model alone, the hybrid models combined with chemical structures and in vitro biological data could enhance the prediction accuracy of human hepatotoxicity [74]. Puri et al. collected preclinical liver biopsy histopathology images for 10 common drugs that presented hepatic necrosis DILI phenotypes and input them into an artificial neural network to develop an AutoML model. This model was able to classify necrotic liver injury patterns accurately with an average precision of 98.6% [75].

Mechanisms of drug action can also be considered during the modeling process. Wu et al. incorporated the mode of action of 333 drugs into the QSAR model, which was divided into active and inactive and yielded a predictive accuracy of 0.711 [76].

### 3.2. Mechanism-Based Prediction

**Figure 3:**
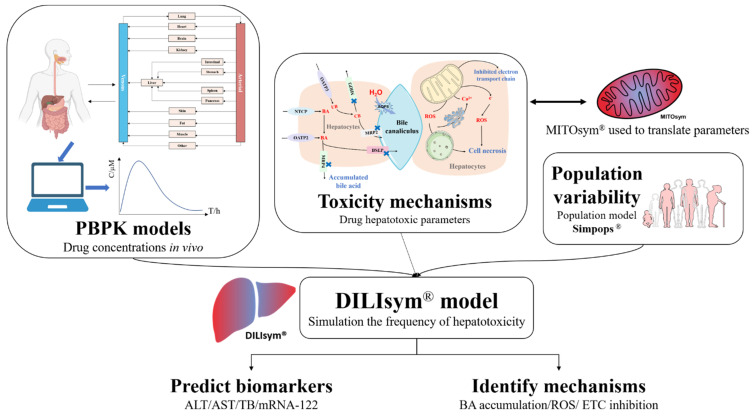
The illustration of DILIsym. DILIsym model integrates physiologically based pharmacokinetic (PBPK) model, hepatotoxic mechanisms of drugs, and population variability to simulate the occurrence and development of drug−induced liver injury (DILI), predicting the time−dependent release of biomarkers into serum and assisting the determination of DILI mechanisms. Mitochondrial dysfunction can be further investigated in MITOsym.

A mechanistic approach that is currently being developed—known as DILIsym—could provide a bottom-up prediction of liver safety liabilities in new drug candidates. It integrates pharmacokinetics exposure, mechanisms of hepatotoxicity, and interpatient variability into the modeling process to demonstrate the frequency and the extent of a new DILI in an average patient or a specific population [77] (Figure 3).

DILIsym can simulate the occurrent process of hepatotoxicity, incorporating submodels for the production of reactive metabolites, and generation of ROS (oxidative stress). Mitochondrial dysfunction can be further investigated in MITO-sym [78], accumulation of toxic bile acids within the hepatocytes, lipotoxicity, as well as hepatocyte regeneration in response to injury [79]. DILIsym can also analyze the interaction between hepatocytes and immune cells and simulate the production of innate immune responses in DILI [80]. Combining the time-concentration profile in specific organs with the dose-effect relationship of each biological process in DILI production assessed with in vitro systems [81], DILIsym will predict the time-dependent death of hepatocytes and hence the time-dependent release of biomarkers into serum [82].

During the modeling process of DILIsym, a physiologically based pharmacokinetic (PBPK) model is created using available parameters related to the drug properties and physiological structures to estimate the time-dependent exposure of the drug in the region of interest. With the development of modeling theory, technology in the engineering field, and the popularization of computer technology and computing software, PBPK modeling techniques have matured considerably since their inception in the early 1930s [83]. The PBPK model mathematically describes the physiological processes, including absorption, distribution, metabolism, and excretion (ADME) of chemicals within the body of an organism through computers [84]. Unlike the classical atrioventricular model, most of the parameters of the PBPK model have physiological significance. Once the parameters are determined, the model can simulate and predict drug disposition in a specific organ or tissue under various conditions. As a result, the PBPK model is recognized as a “bottom-up” model [85]. Due to its superior predictive capability, PBPK models have been applied widely in many fields, including the development of drug candidates the design of clinical trial protocols [86], as well as the prediction of clinical drug-drug interactions [87].

The first drug modeled by DILIsym was APAP where the model generated oxidative stress accounting for APAP overdosed hepatotoxicity. The modeling was used to propose the optimal treatment protocol with N-acetyl cysteine [88]. Subsequently, Smith et al. predicted the clinical risk of hepatotoxicity of ubrogepant, telcagepant, and MK-3207 through DILIsym modeling. Telcagepant and MK-3207 were predicted to cause the rise out of the upper limit of normal ALT or total bilirubin at clinical pharmacologic doses, in accordance with clinical observation. Ubrogepant was predicted to be safe for the liver in all simulated individuals at all efficacious doses and a 10-fold higher amount than the proposed clinical dose, supporting the liver safety profile of ubrogepant in clinical trials [89]. Diane et al. also used DILIsym to compare the potential liver toxicity of oral riluzole tablets versus BHV-0223, a novel sublingual formulation of riluzole. The results suggested that sublingual BHV-0223 had reduced hepatic exposure and, consequently, posed a lower risk of liver toxicity compared with riluzole oral tablets [90].

Several fundamental DILI mechanisms are integrated into DILIsym, including mitochondrial toxicity, bile acid-mediated toxicity, and oxidative stress. By sequentially turning off each of these hepatotoxicity mechanisms and observing the DILI outcome change degree of each alteration, the predominant mechanism of hepatotoxicity can be found [91]. By adjusting the parameters associated with hepatotoxicity mechanisms, the ultimate change in DILI outcome can be observed (i.e., parameter sensitivity analyses) to facilitate the conduct of in vitro assays. Therefore, DILIsym can be used to explore the profound effect on the human hepatotoxicity of specific drugs. Tolvaptan, an anti-hyponatremia drug for treating autosomal dominant polycystic kidney disease (ADPKD) is a good example to demonstrate such a feature by DILIsym. Tolvaptan had received a black box warning regarding hepatotoxicity. In clinical practice, ADPKD patients are assumed to be more susceptible to tolvaptan-induced liver injury based on the evidence that no signals of liver safety emerged during prior clinical trials or clinical use of tolvaptan in non-ADPKD patient populations. James et al. used DILIsym to simulate the impact of reduced biliary efflux, which was one of the common manifestations in ADPKD patients on tolvaptan-associated hepatotoxicity. They altered the biliary excretion parameters Vmax of tolvaptan and DM-4103, the main metabolite of tolvaptan, and observed the resultant changes in the pharmacokinetics of tolvaptan and DM-4103, bile acid, mitochondrial homeostasis, and clinical biomarker measures. The results showed that a reduction in the biliary excretion V_max_ of tolvaptan had a minor impact on tolvaptan pharmacokinetics and hepatotoxicity, but that of DM-4103 resulted in marked hepatic accumulation of DM-4103 and bile acids, reductions in hepatic ETC activity and ATP concentrations and increase in hepatotoxicity plasma biomarkers. This DILIsym model supported the hypothesis that impaired biliary efflux increased susceptibility to tolvaptan-associated hepatotoxicity observed in patients with ADPKD, and MRP2 played a more prominent role in tolvaptan-associated liver injury owing to the inhibition of DM-4103 on MRP2 [92].

## 4. Discussion

According to the General Practice Research Database (GPRD), the crude incidence of drug hepatotoxicity in the UK is 2.4 cases per 100,000 people per year [1]. Delayed diagnosis and treatment of DILI have resulted in an increased burden on the healthcare system, as well as causing preventable morbidity and mortality among patients who rely on these drugs. In addition to clinical consequences, DILI is also a major reason for drug withdrawal and a cost driver for pharmaceutical companies. As such, there is an urgent need to improve the capability to predict DILI risks at the early stage of drug development. The pharmaceutical industry has increasingly turned to in silico modeling, and the technique could hold the key to better risk prediction.

Knowledge-based predictive models use the computational algorithm and specific descriptors to develop the relationship between the drug properties and DILI outcome and further predict the DILI risk. The most challenging problem of knowledge-based predictive models is the lack of DILI annotation datasets, which can be defined as a comprehensive collation of drug information, including the hepatotoxicity descriptions from available data, dosing regimen, basic properties, related study results, and the risk classification schemes of DILI into a single database. There are various public DILI annotation datasets with different drugs and content, for example, the LiverTox Dataset [93], the LTKB dataset [94], and DILIst [95]. However, these datasets are still several orders of magnitude smaller than the benchmark datasets used in drug discovery [96]. This is the bottleneck that restricts widespread validation and application of knowledge-based predictive models to the development of new chemical entities. Ma et al. established a property augmentation approach to include massive training data and significantly improved the predictive accuracy to 81.4% using cross-validation [97], indicating the importance of the data size for the predictive results. In this case, more effort should be made with the extension of DILI annotation datasets. For example, some drugs withdrawn from the world market or only listed in one country can be embraced into the research scope. In addition, the risk judgment of drugs in datasets can be more concrete and precise by incorporating information both from the literature and clinical outcomes.

In addition, DILI is often simplified to a qualitative classification problem in the case of computational predictions. This approach, however, does not provide sufficient information on essential factors such as dose dependency or affected patient population during model development. Consequently, the practical applicability of such models is limited. Mechanism-based prediction algorithms such as DILIsym can combine cell assays, PBPK modeling, and interindividual variation into a single model to predict the time course of drug hepatotoxicity. The ability to simulate different dosing regimens and to predict the corresponding DILI risk of each regimen can help to clarify some fundamental clinical questions, including the dose-dependency of a particular drug and determination of safety margin can be undertaken through dose escalations in the virtual subjects within DILIsym [98]. Additionally, hepatotoxicity prediction using DILIsym can circumvent the problem of species differences in preclinical DILI research, especially bile acid-associated liver injury [99,100]. However, the DILIsym model does not include all mechanisms of hepatotoxicity, primarily the immune-related liver injury as previously described. This could potentially lead to a certain degree of underestimation of the risk of DILI. In addition, the DILIsym model cannot mimic various special populations, especially the diseased population that is most vulnerable to DILI. Lastly, most in silico predictive models of DILI, exclude information on macromolecules, metallic, and inorganic compounds resulting in few predictions of DILI of these drugs. These problems can turn into the main research interests of DILI prediction in the future.

DILI in China accounts for about 20% of the hospitalization rate of acute liver injury [101], which could be attributed to both western medicine and traditional Chinese medicine (TCM) [24]. This highlights the importance and urgent need to predict TCM-induced liver injury in China and other regional countries that utilize TCM as part of the healthcare system. Huang et al. used the validated QSAR model that was based on the Liver Toxicity Knowledge Base to investigate the hepatotoxic potential of identified ingredients in the Traditional Chinese Medicine Database of Taiwan. The result showed 74.8% of 9160 unique chemicals possessed hepatotoxic potential, with high hepatotoxic potential for 100 chemical ingredients [102]. The results called for immediate attention to conducting comprehensive assessments of TCM-induced liver injury, especially using mixed model approaches that have been proposed to improve the predictive accuracy of computational models of TCM-induced toxicity. Typically these models would integrate physicochemical data, observational data on drug toxicity, and biological information into a single database for model development using different machine-learning methods [103]. For example, Li et al. successfully used an SVM classifier to delineate the relationship between in vitro hepatotoxic benchmark concentrations and in vivo AUC of training sets, followed by estimating the in vivo plasma profile based on the cytotoxicity data of natural products derived from traditional Chinese medicines (NP-TCMs) of interest by in vitro–in vivo extrapolation (IVIVE) relationship. Then, the oral dosing schedules of NP-TCMs were predicted using PBPK modeling reversely to help safety assessment of TCM-induced liver injury [104].

Theoretically, all subjects who received a drug share the same risks of experiencing hepatotoxic drugs. However, certain populations will be more vulnerable to hepatotoxic drugs. Applying in silico models mentioned above in the analyses of DILI risk factors among vulnerable patient groups is of great significance for DILI research. Related genes have always been studied to understand the pathogenesis of specific DILIs. Moore et al. used machine learning approaches, including multivariate adaptive regression splines (MARS), multifactor dimensionality reduction (MDR), and logistic regression to investigate single-nucleotide polymorphism (SNP)–SNP interaction as a potential DILI risk factor [105]. Moreover, gender has also played an important role in IDILI. Women are more susceptible to hepatotoxicity from certain drugs, such as minocycline and methyldopa [106]. In addition, environmental factors leading to drug-induced liver injury cannot be ignored. Excessive drinking may increase the risk of DILI caused by duloxetine, APAP, methotrexate, and isoniazid [24]. However, few DILI predictions focus on vulnerable populations, which can be one of the important issues for further study.

In conclusion, we have introduced two main types of in silico models for DILI prediction: knowledge-based models using the computational algorithm and mechanism-based models using DILIsym, which could be used to evaluate the risk of drug-associated liver adverse events in both clinical settings and drug development processes.

## Data Availability

No new data were created or analyzed in this study. Data sharing is not applicable to this article.
